# RETRACTION: Enhanced Wound Recovery After Surgery Care in Patients With Total Knee Arthroplasty: A Meta‐Analysis

**DOI:** 10.1111/iwj.70535

**Published:** 2025-04-18

## Abstract

**RETRACTION:** WangL. and LinZ. , “Enhanced Wound Recovery After Surgery Care in Patients With Total Knee Arthroplasty: A Meta‐Analysis,” International Wound Journal 21, no. 1 (2024): e14672, 10.1111/iwj.14672.PMC1083091642052985

The above article, published online on 31 January 2024, in Wiley Online Library (http://onlinelibrary.wiley.com/), has been retracted by agreement between the journal Editor in Chief, Professor Keith Harding; and John Wiley & Sons, Ltd. Following an investigation by the publisher, all parties have concluded that this article was accepted solely on the basis of a compromised peer review process. The editors have therefore decided to retract the article. The authors did not respond to our notice regarding the retraction.

**RETRACTION:** L. Wang and Z. Lin , “Enhanced Wound Recovery After Surgery Care in Patients With Total Knee Arthroplasty: A Meta‐Analysis,” International Wound Journal 21, no. 1 (2024): e14672, 10.1111/iwj.14672.PMC1083091642052985

The above article, published online on 31 January 2024, in Wiley Online Library (http://onlinelibrary.wiley.com/), has been retracted by agreement between the journal Editor in Chief, Professor Keith Harding; and John Wiley & Sons, Ltd. Following an investigation by the publisher, all parties have concluded that this article was accepted solely on the basis of a compromised peer review process. The editors have therefore decided to retract the article. The authors did not respond to our notice regarding the retraction.

